# Dual Targeting EGFR and STAT3 With Erlotinib and Alantolactone Co-Loaded PLGA Nanoparticles for Pancreatic Cancer Treatment

**DOI:** 10.3389/fphar.2021.625084

**Published:** 2021-03-19

**Authors:** Shihui Bao, Hailun Zheng, Jinyao Ye, Huirong Huang, Bin Zhou, Qing Yao, Guangyong Lin, Hailin Zhang, Longfa Kou, Ruijie Chen

**Affiliations:** ^1^ Department of Pharmacy, The Second Affiliated Hospital and Yuying Children’s Hospital of Wenzhou Medical University, Wenzhou, China; ^2^ Wenzhou Municipal Key Laboratory of Pediatric Pharmacy, Wenzhou, China; ^3^ The Second School of Medicine, Wenzhou Medical University, Wenzhou, China; ^4^ School of Pharmaceutical Sciences, Wenzhou Medical University, Wenzhou, China; ^5^ Department of Children’s Respiration Disease, The Second Affiliated Hospital and Yuying Children’s Hospital of Wenzhou Medical University, Wenzhou, China

**Keywords:** nanoparticles, STAT3, EGFR, synergistic effect, pancreatic cancer

## Abstract

Pancreatic cancer (PC) is one of the most common malignancies and also a leading cause of cancer-related mortality worldwide. Many studies have shown that epidermal growth factor receptor (EGFR) is highly expressed in PC, which provides a potential target for PC treatment. However, EGFR inhibitors use alone was proven ineffective in clinical trials, due to the persistence of cellular feedback mechanisms which foster therapeutic resistance to single targeting of EGFR. Specifically, the signal transducer and activator of transcription 3 (STAT3) is over-activated when receiving an EGFR inhibitor and is believed to be highly involved in the failure and resistance of EGFR inhibitor treatment. Therein, we hypothesized that dual inhibition of EGFR and STAT3 strategy could address the STAT3 induced resistance during EGFR inhibitor treatment. To this end, we tried to develop poly (lactic-co-glycolic acid) (PLGA) nanoparticles to co-load Alantolactone (ALA, a novel STAT3 inhibitor) and Erlotinib (ERL, an EGFR inhibitor) for pancreatic cancer to test our guess. The loading ratio of ALA and ERL was firstly optimized *in vitro* to achieve a combined cancer-killing effect. Then, the ALA- and ERL-co-loaded nanoparticles (AE@NPs) were successfully prepared and characterized, and the related anticancer effects and cellular uptake of AE@NPs were studied. We also further detailly explored the underlying mechanisms. The results suggested that AE@NPs with uniform particle size and high drug load could induce significant pancreatic cancer cell apoptosis and display an ideal anticancer effect. Mechanism studies showed that AE@NPs inhibited the phosphorylation of both EGFR and STAT3, indicating the dual suppression of these two signaling pathways. Additionally, AE@NPs could also activate the ROS-p38 axis, which is not observed in the single drug treatments. Collectively, the AE@NPs prepared in this study possess great potential for pancreatic cancer treatment by dual suppressing of EGFR and STAT3 pathways and activating ROS-responsive p38 MAPK pathway.

## Introduction

Pancreatic cancer (PC) remains a highly fatal disease with dismal prognosis even after surgical resection, and the 5 years survival rate of PC patients is only approximately 10% in USA (Siegel et al., 2020). According to the International Agency for Research on Cancer (IARC), PC has been the seventh leading cause of cancer death worldwide, accounting for many death (n = 432,000) as cases (n = 459,000) (Bray et al., 2018). Recently, great efforts have been made in molecular targeted therapies to inhibit constitutively activated kinase pathways in PC, which significantly improve the progression-free survival and overall survival (Philip and Lutz, 2015). Many studies showed that epidermal growth factor receptor (EGFR) signaling is significantly upregulated in PC (Guo et al., 2016; Blasco et al., 2019; Engle et al., 2019). Therefore, EGFR could be a rational therapeutic target for PC therapy. Erlotinib (ERL), an FDA-approved EGFR inhibitor, has shown therapeutic effect in the treatment of PC when used in combination with gemcitabine (Sinn et al., 2017; Halfdanarson et al., 2019). However, drug resistance induced by EGFR inhibitors invariably limits the treatment response of oncogene-addicted cancer cells (Chong and Janne, 2013; Nagathihalli et al., 2018). Acquired resistance occurs in patients who initially benefit from EGFR targeted therapies and later prevent them from having a response with clinical benefits. Increased understanding of the detailed mechanisms about acquired resistance during EGFR therapy would be of clinical importance. Specifically, the activation of a bypass signaling pathway, signal transducer and activator of transcription 3(STAT3) pathway which has been proved to be an important oncogenic factor that regulates a plethora of biological functions, including cell differentiation, angiogenesis, proliferation, apoptosis, and inflammation, has been elucidated in patients that are resistant to EGFR treatment (Lankadasari et al., 2018; Nagathihalli et al., 2018; Dosch et al., 2020). In addition, it was found that the overexpression of STAT3 contributed to drug resistance in various oncogene-addicted cancers (Wen et al., 2015; Zhao et al., 2016; He et al., 2018). Based on these results, Ji et al. found that sodium cantharidate that could deregulate STAT3 signaling successfully abrogated EGFR inhibitor resistance in osteosarcoma (Ji and He, 2019). In another study, Hu et al. showed that berbamine enhanced the efficacy of gefitinib, a classic EGFR inhibitor, by suppressing STAT3 signaling in PC (Hu et al., 2019). These findings further confirmed the role of EGFR-STAT3 feedback activation loop in EGFR treatment resistance. Additionally, PC is characterized by a robust desmoplastic environment, which limits the uptake of drugs. Austin R. Dosch et al. found that the combined inhibition of Src/EGFR and STAT3 signaling induced stromal remodeling to improve survival in PC (Dosch et al., 2020). Therefore, double blocking of both EGFR and STAT3 could be an effective strategy for PC therapy.

Nano-drug delivery systems (NDDS), including biodegradable nanoparticles, liposomes, micelles, etc., have been utilized as a potent formulation strategy in various biomedical applications to improve therapeutic outcomes (Kou et al., 2018; Yao et al., 2018; Yao et al., 2020a; Moradi Kashkooli et al., 2020; Shao et al., 2020). NDDS could improve the stability and solubility of therapeutics, as well as optimize the *in vivo* behavior by prolonging circulation time, enhanced penetration and retention effect, and reduced side effects of drugs. In addition, NDDS could provide simultaneous drug loading and deliver two or three, and even more drugs for combination treatment. For example, Yao et al. developed a nano-micelles for both paclitaxel and curcumin co-delivery to enhance cancer cell killing effect and circumvent resistance (Yao et al., 2017). We also prepared a multifunctional liposome loading doxorubicin and sorafenib to destruct the intracellular redox homeostasis for enhanced anticancer therapy (Kou et al., 2020c). Consequently, NDDS may provide a feasible strategy for PC combination drug therapy.

**SCHEME 1:**
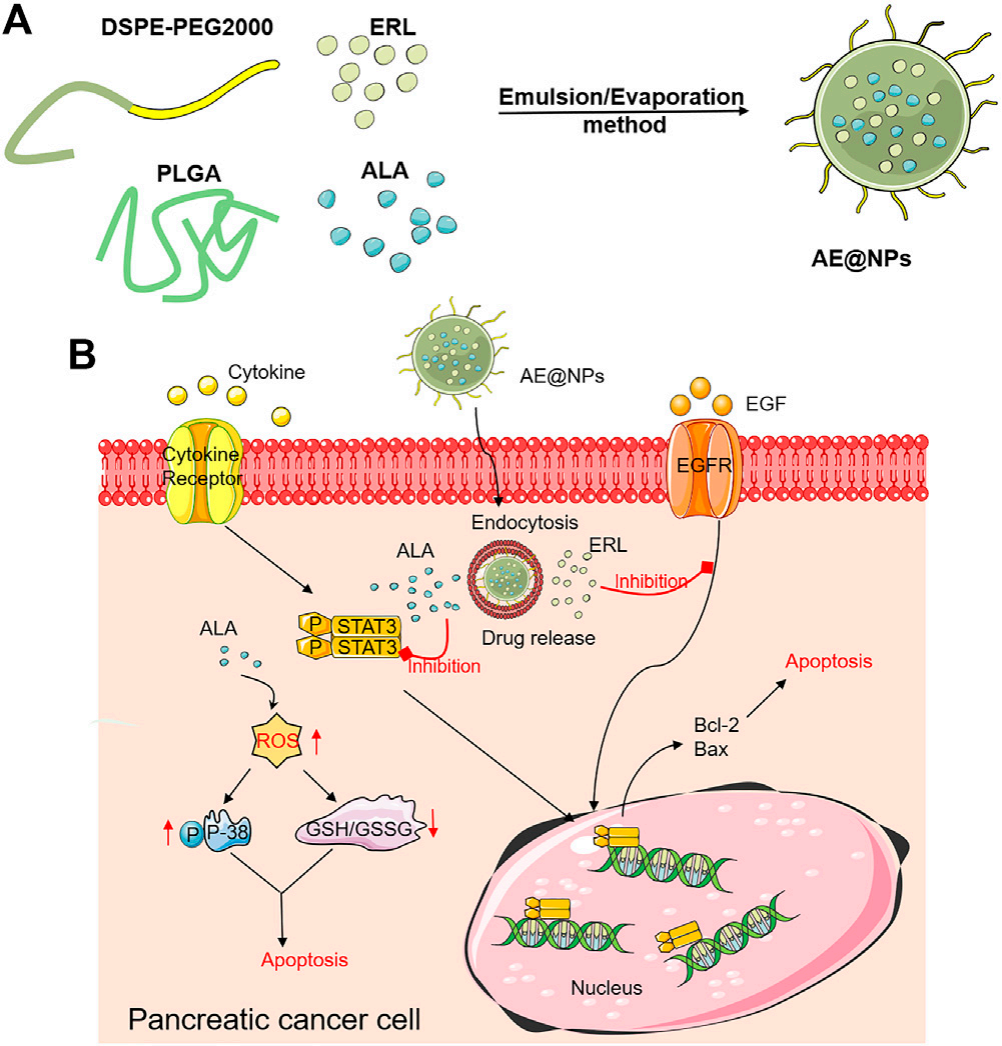
Schematic illustration of the design and application of AE@NPs for enhanced PC treatment. **(A)** The design of ALA and ERL co-loaded nanoparticles (AE@NPs). **(B)** The action mechanisms of AE@NPs in PC cells. AE@NPs firstly entered cells via endocytosis and released ALA and ERL within PC cells. The released ALA could inhibit the activation of STAT, and ERL could inhibit the EGFR signal pathway. In addition, AE@NPs-induced upregulated ROS could activate p38-MAPK pathway. These effects collectively contributed to the enhanced cell-killing, anti-proliferation, and anti-metastasis effects of AE@NPs in PC cells.

In this study, we would like to employ the co-delivery strategy to dual-block EGFR and STAT3 by NDDS for effective and enhanced PC therapy (Scheme 1). Erlotinib (ERL), one of the first-generation FDA-approved small molecule EGFR inhibitors used in the treatment of lung and pancreatic cancer, was chosen; a major constituent of Inula helenium Alantolactone (ALA) which was confirmed to possess STAT3 inhibition property in our previous study (Zheng et al., 2019) and also other studies (Chun et al., 2015; Cui et al., 2018), was selected as the STAT3 inhibitor. Firstly, the ratio of ERL and ALA was screened for the best therapeutic outcome. Then, poly (lactic-coglycolic acid) (PLGA) nanoparticles co-loading ERL and ALA with the optimized ratio (AE@NPs) were prepared and characterized. The uptake efficiency and cell-killing effect were then evaluated. We systemically investigated the underlying molecular mechanisms for the anticancer treatment and found that, besides EGFR and STAT3 inhibition, the ROS-p38 axis was also activated, which was also related to the enhanced therapeutic outcome. This study provided a viable and potential approach for effective PC treatment.

## Materials and Methods

### Materials

Human pancreatic cancer cell lines (PANC-1 and Patu8988T) were obtained from Shanghai Institute of Biosciences and Cell Resources Center (Chinese Academy of Sciences, Shanghai, China). PANC-1 and Patu8988T cells were grown in Roswell Park Memorial Institute (RPMI)-1640 media (Gibco) with 10% FBS, 100 units/mL penicillin, and 100 μg/ml streptomycin at 37 C in a humidified incubator with 5% CO_2_.

Erlotinib and Alantolactone were obtained from Aladdin (Shanghai, China). PLGA (38,000 MW) was bought from Jinan Daigang Biological Engineering Co. Ltd (Jinan, China). Poly (vinylalcohol) (PVA; 20,000–30,000 MW) was purchased from Acros Organics (New Jersey, USA). DSPE-PEG2000 was bought from AVT (Shanghai) Pharmaceutical Co., Ltd. Methyl thiazolyl tetrazolium (MTT) was obtained from Aladdin (Shanghai) Biochemical and Technology Co., Ltd. The antibodies against p-STAT3, STAT3, β-Actin, Bax, Bcl-2 were purchased from Cell Signaling Technology (Danvers, MA, USA). Antibodies of p-EGFR, EGFR was purchased from Santa Cruz Biotechnology Inc (Dallas, TX, USA). The horseradish peroxidase (HRP)-conjugated goat anti-mouse IgG and HRP-conjugated goat anti-rabbit IgG were obtained from Biosharp Biotechnology. Other chemicals or reagents were of analytical grade.

### Preparation of AE@NPs

A modified solvent extraction/evaporation technique was used to prepare AE@NPs (Kou et al., 2017; Zhang et al., 2019; Zheng et al., 2020). In brief, the appropriate amount of PLGA, DSPE-PEG2000, ALA, and ERL was dissolved in dichloromethane (DCM), and then 1% (v/v) PVA solution was added. The formed mixture was sonicated under a probe sonication (JY92-2D, Scientz, China). The formed emulsion was continuously stirred for 6 h to evaporate the solvent DCM, and the solution was then centrifuged (20,000 rpm, 30 min) to achieve the nanoparticle sedimentation. The nanoparticles were then washed with double-distilled water three times, and freeze-dried for following characterization and application. ERL-loaded nanoparticles (E@NPs) were prepared as control by removing ALA in the preparation process. When coumarin 6 (C6) was used a probe, ERL was replaced by C6 to prepare C6-labelled nanoparticles (C6@NPs or C6A@NPs).

### Characterization of AE@NPs

A Nano Zetasizer (Litesizer 500, Anton Paar) was used to measure the particle size, size distribution, and zeta potential of AE@NPs. The changes in particle size and polydisperse index (PDI) were monitored to evaluate the stability of nanoparticles. The morphologies of nanoparticles were visualized by Transmission electron microscopy (TEM) (1200EX, JEM). Briefly, the nanoparticles were dropped onto a carbon-coated copper grid and then counterstained with 0.5% phosphotungstic acid. After that, the sample was dried and ready for TEM measurement.

The drug load (DL) and encapsulation efficacy (EE) were measured as previously (Zhang et al., 2015; Han et al., 2019; Kou et al., 2020a). Briefly, a Sephadex G50 column was prepared and used to remove the unloaded free drug by elution. The nanoparticles were destroyed by excess acetonitrile, and the concentration of drugs was determined by high performance liquid chromatography (HPLC) method. The DL and EE were calculated by the following equations: DL (%) = (weight of the drug in NPs/weight of the drug-loaded NPs) × 100%; EE (%) = (weight of the drug in NPs/weight of the drug added) × 100%.

The *in vitro* release kinetics of drugs from NPs were investigated by a modified dialysis method (D'Souza and DeLuca, 2005; Zhao et al., 2020). Briefly, the dialysis bag (8,000–12,000 MW) containing the various formulations were dispersed in phosphate-buffered saline (PBS) (pH 7.4) containing 1% Cremophor EL (w/v). Specific volume samples were taken at designated intervals for analysis by HPLC, and the same volume of fresh medium was replenished.

### MTT Assay

MTT assay was performed according to the reported procedure (Yao et al., 2020b; Yao et al., 2020c; Kv et al., 2020). The human pancreatic cancer cells were plated 5,000 cells per well in a 96-well plate and cultured in RPMI 1640 medium containing 10% heat-inactivated FBS for 12 h. The cells were then treated with various drug treatments for 24 h. Following that, the MTT solution was added and incubated at 37°C for 4 h. The formed formazan was dissolved in 100 µL DMSO, and the absorption was then determined by a microplate reader. The IC50 was calculated by GraphPad Software 8.0 (GraphPad Software, Inc.). The combination index (CI) was calculated based on the following equation: CI = a/A+b/B. a is the IC50 of ERL in combination use, and b is the IC50 of ALA in combination use; A is the IC50 of ERL when used alone, B is the IC50 of ALA when used alone. CI < 0.9 suggests a synergistic effect; 0.9 ≤ CI ≤ 1.1 suggested an additive effect; CI > 1.1 suggested an antagonistic effect.

### Wound-Healing Migration Assay

The cells were seeded into 6-well plate and allowing grow up to 90% confluence. Then, the cell monolayers were scratched with a sterile 10 μL pipette to generate a straight wound area. The cells were incubated with different drug treatments and photographed by microscope at 0 h and 36 h after treatments. Each experiment was done in triplicates. The migration rate was determined using the following formula:

$$\text{Migration rate}\left(\text{\%}\right)=\frac{W_{0h}-W_{36h}}{W_{0h}}\times 100\text{\%}\text{.}$$

The wound width at 0 h was labeled as *W*_0h_, and the width of would at 36 h was labeled as *W*_36h_.

### Clonogenic Assay

The cells were seeded into 6-well plates at a density of 500 cells/well and cultured overnight. The cells were treated with different drug treatments for 24 h, and then the fresh medium was added into each well to replace the previous medium and incubated for one week. Colonies were fixed 4% paraformaldehyde and washed with phosphate-buffered saline (PBS) twice. The fixed cells were stained with 1% crystal violet for 10 min at room temperature and photographed. After imaging, a lysis buffer was used to dissolve the stains, and the absorbance at 630 nm was measured for quantitative analysis. Each experiment was done in triplicates.

### Hoechst 33258 Staining

The apoptosis of pancreatic cancer cells was detected by Hoechst 33258 assay. Cells were firstly seeded in 6-well plates and incubated with various drug treatments for 18 h. Then, the cells were washed with PBS and fixed by 4% paraformaldehyde at room temperature for 15 min. The fixed cells were washed with PBS and incubated with Hoechst 33258 for 20 min. The cells were visualized by a fluorescence microscope, and the apoptotic features of the cells were evidenced by the staining of cell nuclei with H33258, which indicates chromatin condensation.

### Cellular Uptake Assay

Cellular uptake and intracellular distribution of NPs were studied by confocal laser scanning microscopy and fluorescence microplate reader. Briefly, PANC-1 and Patu8988T were seeded in 6-well plates overnight. Then, coumarin-6 (C6) solution and C6-labelled nanoparticles (C6@NPs or C6A@NPs) were added into the cultured cells and incubated for pre-determined time at 37°C. Following that, the cells were washed twice with PBS and immobilized by 4% paraformaldehyde for 15 min at room temperature. The fluorescence images were recorded by a confocal laser scanning microscope (Nikon Corporation, Tokyo, Japan), and the fluorescence intensity was measured by a fluorescent microplate reader (Infinite 200Pro, Tecan i-control) by setting the excitation/emission wavelengths at 466 nm/504 nm.

### Western Blotting

Western blot analysis was performed using standard methods. Pancreatic cancer cells were seeded in 6-well plates and incubated with a designated concentration of drugs for 24 h. The cells were washed with PBS and harvested using cell protein lysate buffer, and the protein concentration was determined by bicinchoninic acid assay (BCA assay). The proteins were then run on 10% sodium dodecyl sulfate-polyacrylamide gel (SDS-PAGE) and transferred onto a polyvinylidene fluoride (PVDF) membrane. After blocked with 5% skim milk, the membrane was incubated with primary antibodies as indicated for 12 h at 4°C. Following washing three time, the membranes were incubated with the relevant secondary antibodies, and the detection using enhanced chemiluminescence (ECL) solution was performed.

### Flow Cytometry Analysis

Apoptosis was also determined using an apoptosis detection Kit (BD Biosciences, USA). The PANC-1 and Patu8988T cells were plated on 6-well plates and treated with the indicated drug for 24 h. Cells were collected, washed twice with ice-cold PBS, and resuspended in binding buffer containing Annexin V-FITC/PI according to the instructions of the apoptosis Kit. The stained cells were further analyzed by flow cytometry.

### Measurement of ROS Generation in PC Cells

Cellular ROS contents were measured by flow cytometry and fluorescence microscopy. Briefly, cells were seeded into 6-well culture plates and incubated overnight for attachment. The cells were then treated with different drug treatments. After treatment and wash, 10 μM DCFH-DA (Beyotime Biotech, Nantong, China) was used to detect the generated ROS in cells, and the DCF fluorescence was analyzed using flow cytometry and recorded by fluorescence microscopy.

### Statistical Analysis

Statistical analysis was conducted with GraphPad Software 8.0 (GraphPad Software, Inc.). The results are expressed as mean ± SD. The difference between the groups was identified by Student's t test or ANOVA followed by Tukey’s multiple comparison test. If the *p*-value was less than 0.05, it would be considered statistically significant.

## Results and Discussion

### Preparation and Characterization of AE@NPs

Firstly, we screened the molar ratio of ALA and ERL for the best combined anticancer effect. As shown in Supplementary Figure S1, the combination use of ERL and ALA displayed significantly increased cell killing ability. When the molar ratio of ALA and ERL was increased 1:2 (ALA: ERL), the cell growth was remarkably suppressed compared to the ALA group, and the CI values were 0.70 and 0.80 in PANC-1 and Patu-8988T cells. The cell killing activity could be further enhanced as ALA: ERL ratio increased, but not that obvious. Also, the CI value did not increase again. In order to balance the synergistic effect and preparation process, 1:2 was selected for the preparation of AE@NPs and following biological application.

**FIGURE 1:**
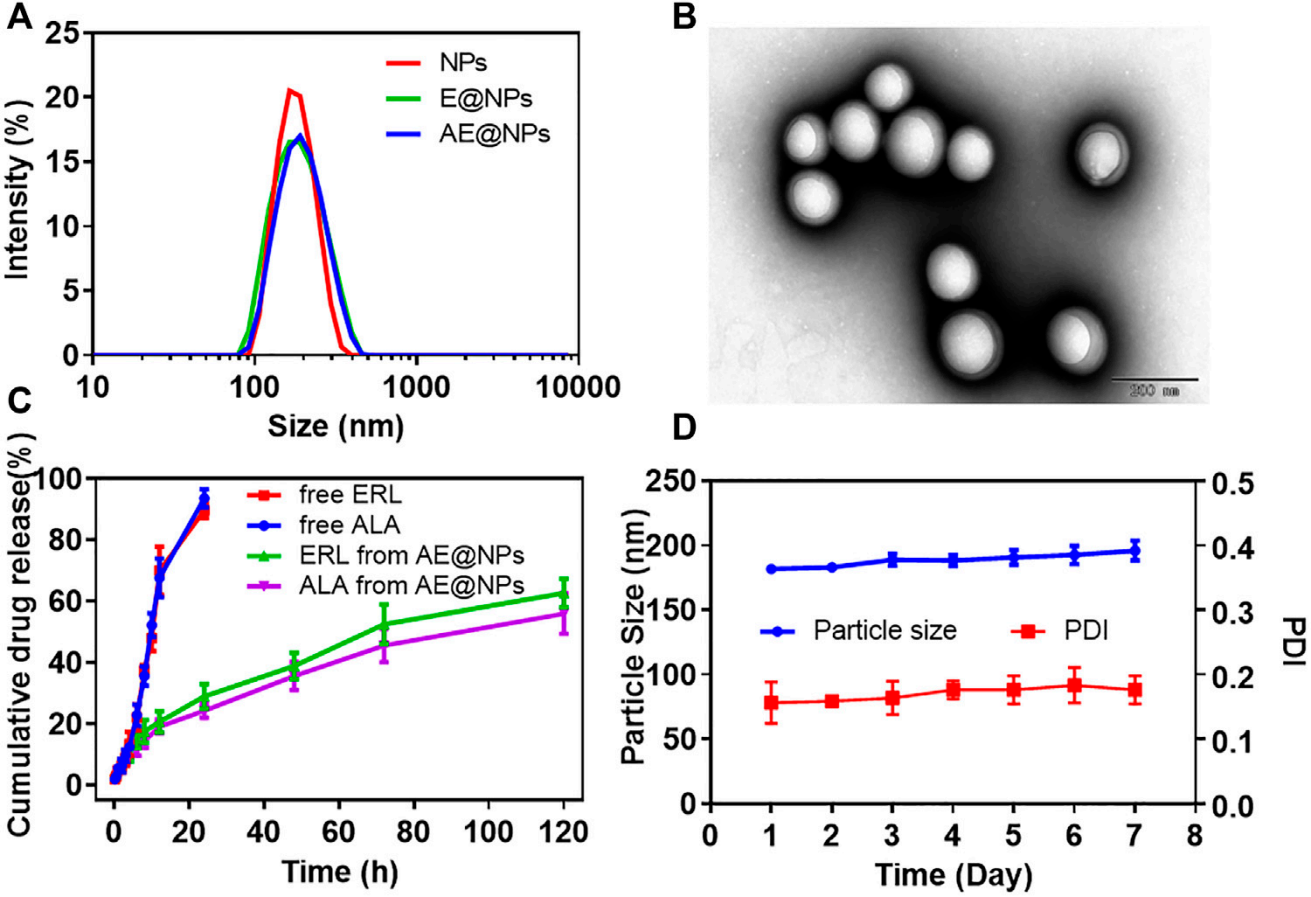
The physicochemical properties of ALA and ERL co-loaded nanoparticles (AE@NPs) were characterized **(A)** Particle size distribution of NPs, E@NPs, and AE@NPs **(B)** TEM image of AE@NPs **(C)** The cumulative drug release of ERL and ALA from free drug solution and AE@NPs **(D)** The changes in particle size and PDI of AE@NPs in pH7.4 PBS at 4 °C in one week. The results were shown as means ± SD (n = 3).

DSPE-PEG2000 was adopted to uniform the size distribution and facilitate the preparation of AE@NPs. Narrow size distribution was presented for each nanoparticle formulation (Figure 1A). The physicochemical properties of nanoparticles were studied (Supplementary Table S1). The dynamic light scattering (DLS) measurement showed the blank nanoparticles were about 178.54 nm, and the drug loading process slightly increased the particle size to 182.69 nm and 185.97 nm for E@NPs and AE@NPs. All PDI values of the nanoparticle samples were lower than 0.2, indicating good dispersibility. The surface potential of nanoparticle was mildly negative and safe for biological applications. The EEs of ERL and ALA were about 90%; the DL of ERL in NPs was higher than 6%, and the DL of ALA in NPs was higher than 3%. Thus, the 1:2 ratio of ALA and ERL, that facilitated a synergistic anticancer effect, was kept in the nanoparticles. TEM images indicated that all nanoparticles were sphere with comparable uniform size (Figure 1B, Supplementary Figures S2A,B). However, the particle size showed in TEM was a little smaller than the value determined by DLS, which might be due to the morphology images showed the dry nanoparticles, while DLS indicated the hydrodynamic diameter of nanoparticles.

**FIGURE 2:**
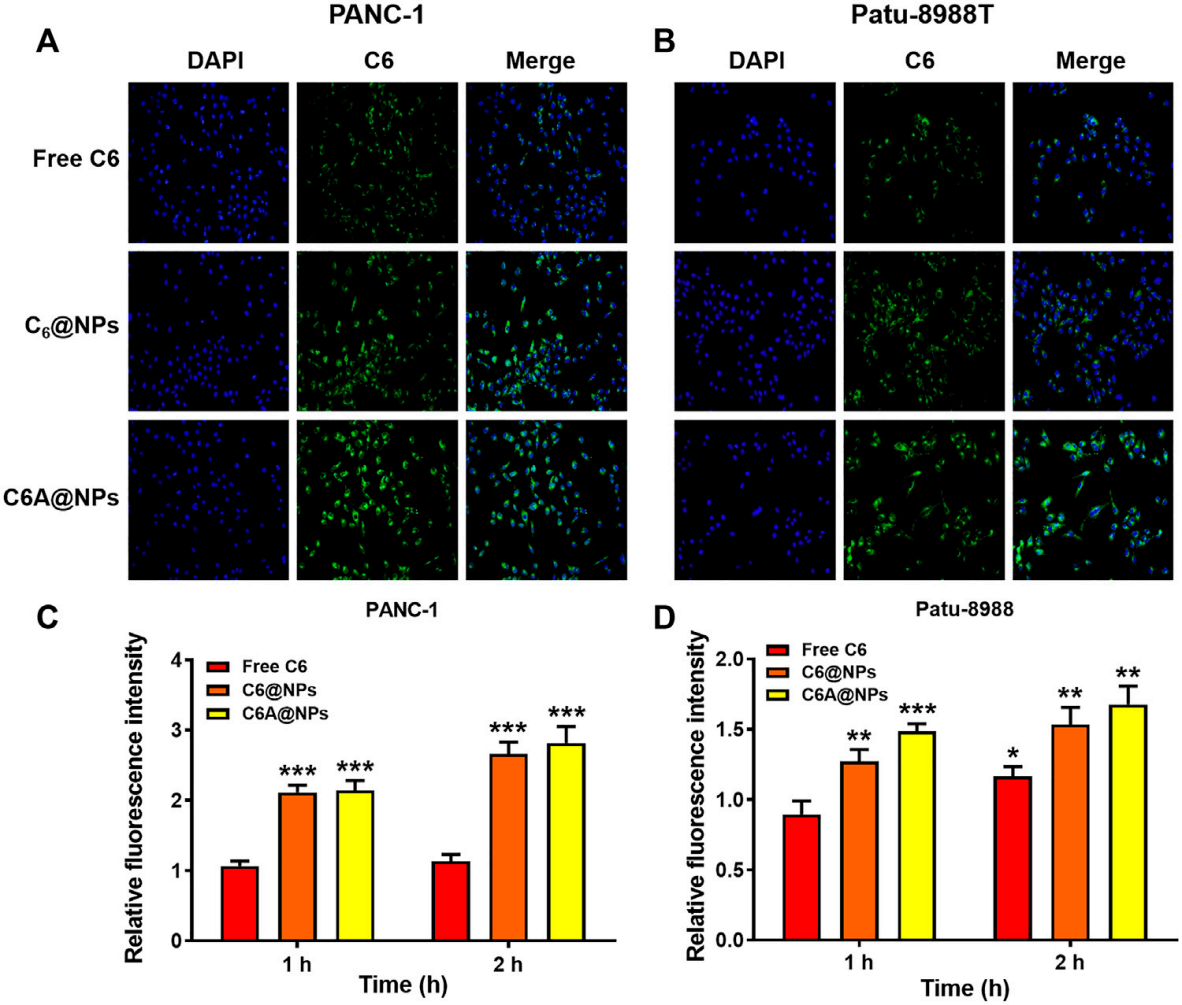
Cellular uptake of C6A@NPs in PANC-1 and Patu-8988T cells **(A)** The fluorescence images (Magnification: ×400) indicated the uptake profiles of drug in each group after 2 h treatment **(B)** Quantitative analysis of cellular uptake efficiency of C6A@NPs by a fluorescence microplate reader. The data were shown as mean ± SD. Experiments were performed in triplicates. *p < 0.05, **p < 0.01, ***p < 0.001, compared to the control group.

The drug release profiles from nanoparticles kept a sustained manner for both E@NPs (Supplementary Figures S2C) and AE@NPs (Figure 1C) in comparison with free drug (Figure 2C). Additionally, the release behaviors of ERL and ALA from AE@NPs were similar, which would be beneficial for the action of combined anticancer activity. In addition, the stability assay suggested that the particle size and PDI values changed very slightly within one week for all nanoparticles (Figures 1D, Supplementary Figure S2D), indicating a good stability for this type of nanoparticles, which was beneficial for the following biological evaluation.

### Cellular Uptake of AE@NPs in PC Cells

We performed uptake assay in PC cells to show the *in vitro* uptake profiles of AE@NPs. It has been reported that endocytosis was usually employed in the uptake process of nanoparticles (Sahay et al., 2010; Kou et al., 2013). The nanoparticles could not only increase the stability and solubility of encapsulated drugs but also shun the quick efflux mediated by ATP-binding cassette (ABC) transporters, including multidrug resistance protein 1 (MRP1), breast cancer resistance protein (BCRP), and P-glycoprotein (P-gp), thereby maintaining their therapeutic effect. The *in vivo* and *in vitro* behaviors of ERL were also affected by ABC transporters, especially BCRP, also named ABCG2 (Elmeliegy et al., 2011). To visualize the intracellular behavior of AE@NPs, coumarin 6 (C6) which shows similar property with ERL was selected as a fluorescence probe, and the uptake process of AE@NPs was monitored by fluorescence microscopy and microplate reader. As shown in Figure 2A (PANC-1) and 2B (Patu-8988T), C6-labelled nanoparticles displayed increased uptake in comparison with the free C6, which might be attributed to the nanoparticle-mediated endocytosis process and the protecting effect of nanoparticles from ABC transporter-mediated drug efflux. Quantitative analysis for the uptake assay were also conducted in both PANC-1 and Patu-8988T cells (Figures 2C,D). The results were consistent with those detected by fluorescence microscope. It should be mentioned that the uptake of C6A@NPs slightly higher than that of C6@NPs in both PANC-1 and Patu-8988T cells. The addition of ALA might affect the function of ABC transporters and then caused the different cellular uptake of C6A@NPs and C6@NPs (Yang et al., 2013; Xu et al., 2019a). Also, the STAT3 inhibition ability of ALA might suppress the cell viability, which in turn facilitated the cellular uptake of nanoparticles.

### Anti-Cancer Efficacy of AE@NPs

**FIGURE 3:**
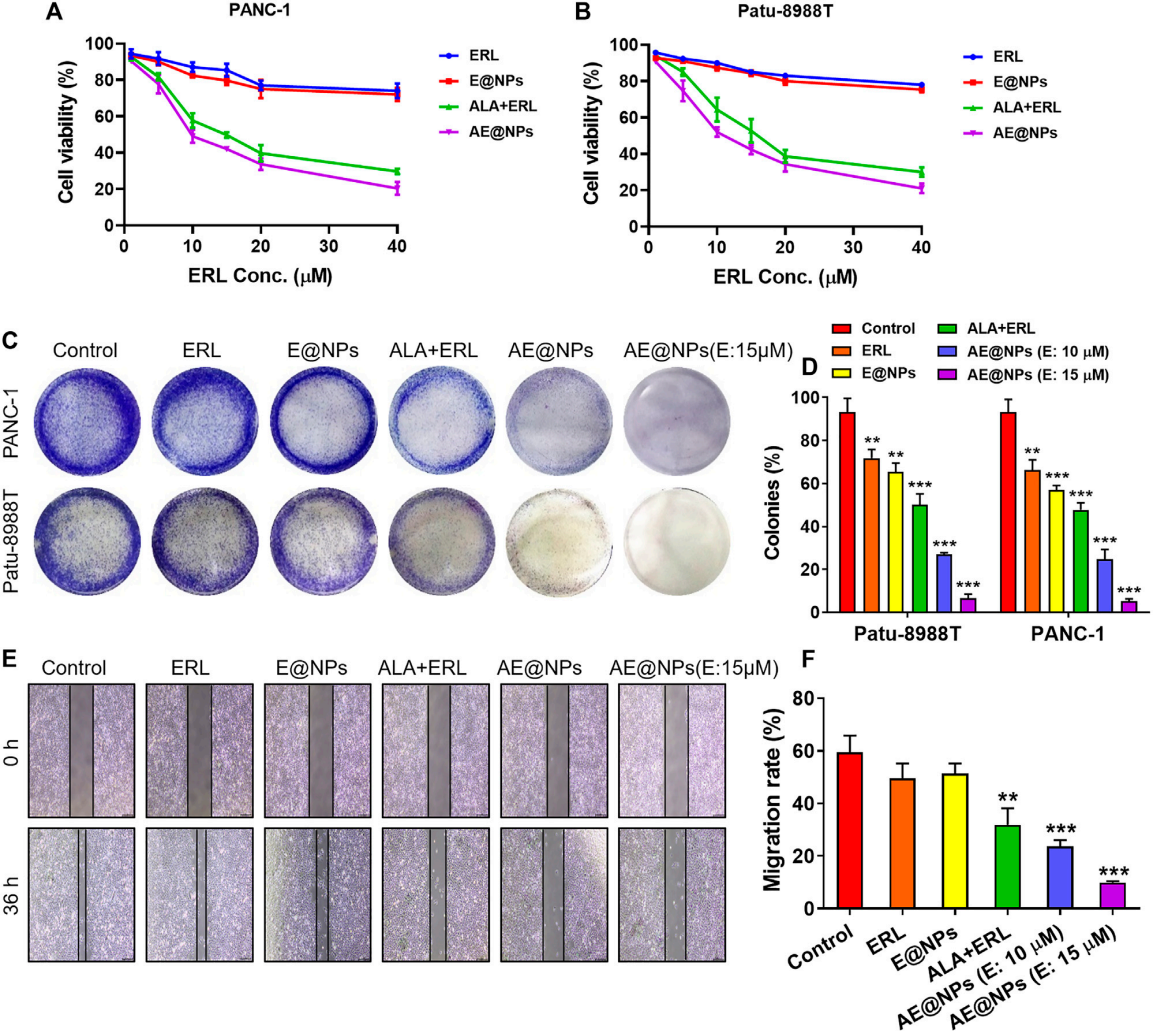
The enhanced anticancer efficacy of AE@NPs in pancreatic cancer cells. MTT assay was conducted to investigate the cell-killing effect of AE@NPs in **(A)** PANC-1 and **(B)** Patu-8988T cells **(C)** Colony forming assay in PANC-1 and Patu-8988T cells. The colonies were fixed with 4% paraformaldehyde and stained with crystal violet **(D)** The colony forming assay was quantified by the absorption of dissolved stain at 630 nm **(E)** The anti-metastasis capability of AE@NPs was studied by wound healing assay and **(F)** the corresponding quantitative analysis. Magnification: ×100. The data were shown as mean ± SD. Experiments were performed was performed in triplicates. *p < 0.05 **p < 0.01, ***p < 0.001, compared to the control group.

EGFR inhibitor therapy could be effective to treat PC by targeting the highly expressed EGFR. However, STAT3 activation induced resistance overset EGFR inhibitor-based PC therapy. To address this issue, a STAT3 inhibitor, ALA, and an EGFR inhibitor, ERL, were loaded together into nanoparticles in this study for enhanced PC treatment. MTT assay was conducted to investigate the anticancer effect of AE@NPs. As shown in Figures 3A,B, the anticancer effect of ALA and ERL was significantly increased compared to that in ERL alone or E@NPs. It was indicated that drug combination dual-targeting both EGFR and STAT3 could enhance the cancer cell killing effect as compared to EGFR inhibitors alone. In addition, AE@NPs also displayed a higher cytotoxicity towards PC cells compared to free ALA plus ERL. The increased cell-killing ability was attributed to the increased uptake (Figure 2).

The anti-proliferation property of AE@NPs was studied by clonogenic assay (Figures 3C,D). The crystal violet-stained clone was recorded by photograph. It was shown that the stain was decreased when drug treatment was applied. The combination use of ERL and ALA always showed higher anti-proliferation property compared to ERL alone due to the dual blocking on EGFR and STAT3 signaling. Dual drug loaded nanoparticles (AE@NPs) showed increased suppression on cell proliferation than free drug combination, which is involved with the increased uptake as presented in Figure 2. Additionally, AE@NPs showed a dose-dependent inhibition on the proliferation of PANC-1 and Patu-8988T cells. The stain was dissolved to measure the absorption at 630 nm for quantitative analysis. As shown in Figure 3D, the quantitative results were consistent with the photos (shown in Figure 3C). In addition, we further performed wound-healing migration assay to investigate the anti-metastasis property of AE@NPs on PANC-1 cells. As shown in Figures 3E,F, the wounds in the control group and free ERL group were almost closed. The combination use of ERL and ALA significantly restricted the closure trend, and AE@NPs displayed the best anti-metastasis ability with a concentration-dependent manner, displaying the lowest migration rate. Taking together, these results suggested that AE@NPs displayed excellent anticancer capacity, including increased cell-killing, anti-proliferation, and anti-metastasis properties.

### The Anticancer Mechanisms of AE@NPs

**FIGURE 4:**
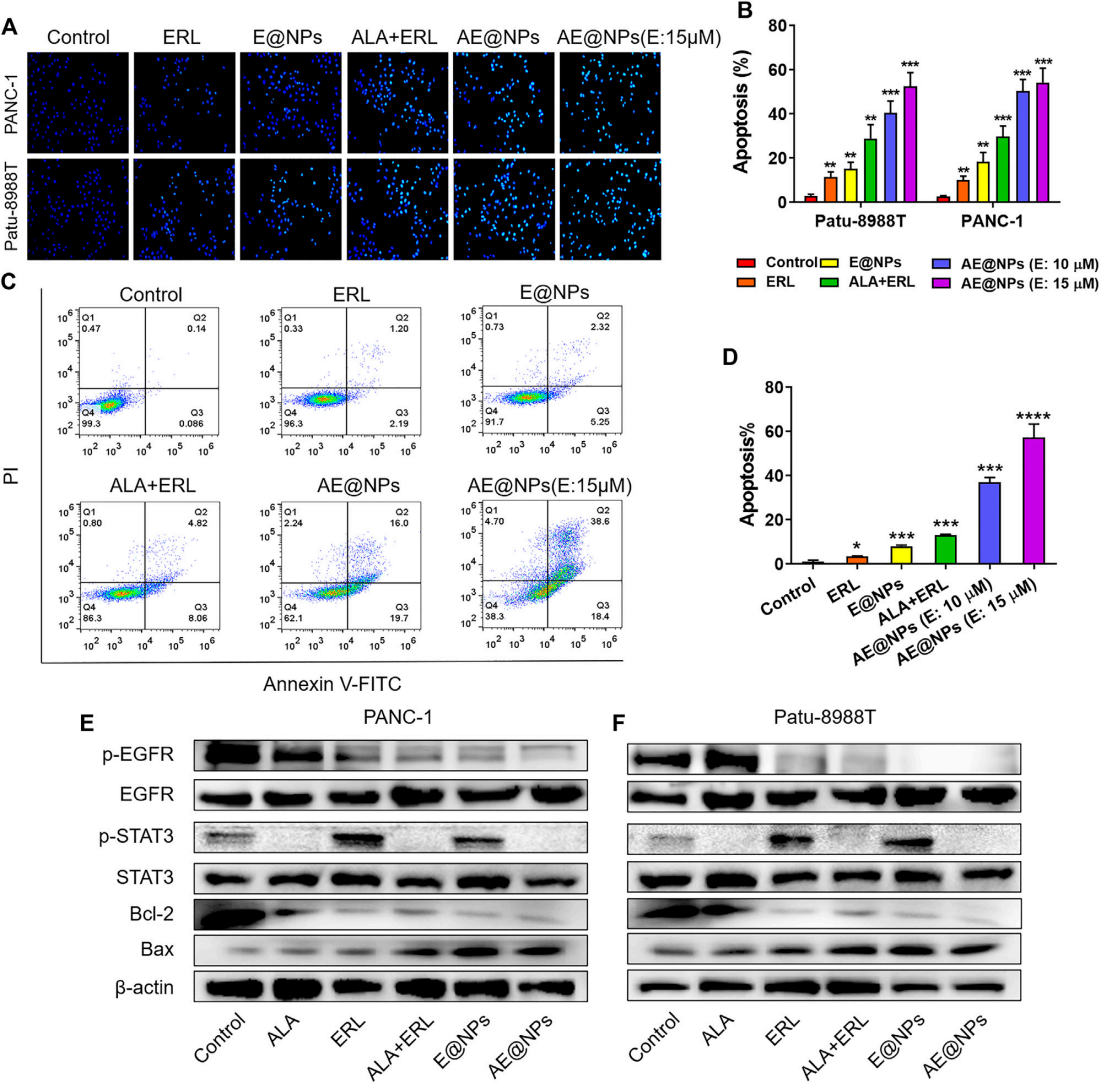
The underlying anticancer mechanisms of AE@NPs **(A)** the apoptosis induced by AE@NPs in PC cells (magnification: ×200) was assessed by Hoechst 33258 staining and also **(B)** quantitatively analyzed **(C)** Annexin V/propidium iodide (PI) staining was used to identify apoptosis, and **(D)** the percent of late-apoptosis was listed **(E)** PANC-1 and **(F)** Patu-8988T were treated with AE@NPs for 18 h, and then the protein levels of *p*-EGFR, EGFR, p-STAT3, STAT3 were determined by western blot. β-actin was used as the internal control. The data were shown as mean ± SD. *p < 0.05 **p < 0.01, ***p < 0.001, ****p < 0.0001, compared to the control group.

Given the enhanced anticancer activity of AE@NPs, we further investigated the underlying mechanisms. Hoechst 33258 staining was firstly used to evaluate the apoptosis in both PANC-1 and Patu-8988T cells after treatments. As shown in Figure 4A, the viable cells in control group showed normal shaped nuclei which were faintly stained. However, AE@NPs treated cells displayed shrunken nuclei with the evidence of chromatin condensation, indicating cell apoptosis. In both cell lines, the combination of free ERL and ALA increased the apoptotic cell ratio, while AE@NPs showed further enhanced pro-apoptosis ability. The quantitative analysis showed a similar trend (Figure 4B). Annexin V-FITC/PI double staining method was further used to evaluate the pro-apoptotic property of AE@NPs. As shown in Figures 4C,D, AE@NPs treatment increased the apoptotic ratio from 0.14% (control group) to 16.0%, suggesting a potent pro-apoptosis property. These results were consistent with the enhanced anticancer results in Figure 3. We further monitored the critical protein level after different drug treatments by using western blot assay. As shown in Figures 4E,F, ALA displayed significant inhibitory effect on the activation of STAT3 evidenced by the decreased expression of p-STAT3; ERL showed distinct p-EGFR inhibition, confirming its own anticancer activity. When ERL and ALA were used together, both signaling pathways (STAT3 and EGFR) were inhibited, resulting in the decreased expression of Bcl-2 and increased expression of Bax. The increased Bax/Bcl-2 ratio suggested the improved apoptosis. When ERL and ALA were encapsulated into nanoparticles (AE@NPs), the pro-apoptosis effect was further increased as compared to that of ERL+ALA group. These results were consistent with the results in Hoechst 33258 assay (Figures 4A,B) and Annexin V-FITC/PI double staining (Figures 4C,D). The underlying anticancer mechanisms of AE@NPs includes the dual blockade of EGFR and STAT3 signaling pathways and nanoparticulate-mediated enhanced cellular uptake. These data suggested that the codelivery of ALA with ERL by nanoparticles could combat drug resistance and provide a novel therapeutic strategy to treat PC by targeting the EGFR and STAT3 pathways.

### ROS-p38 Axis Involved in the Enhanced Anticancer Efficacy of AE@NPs

**FIGURE 5:**
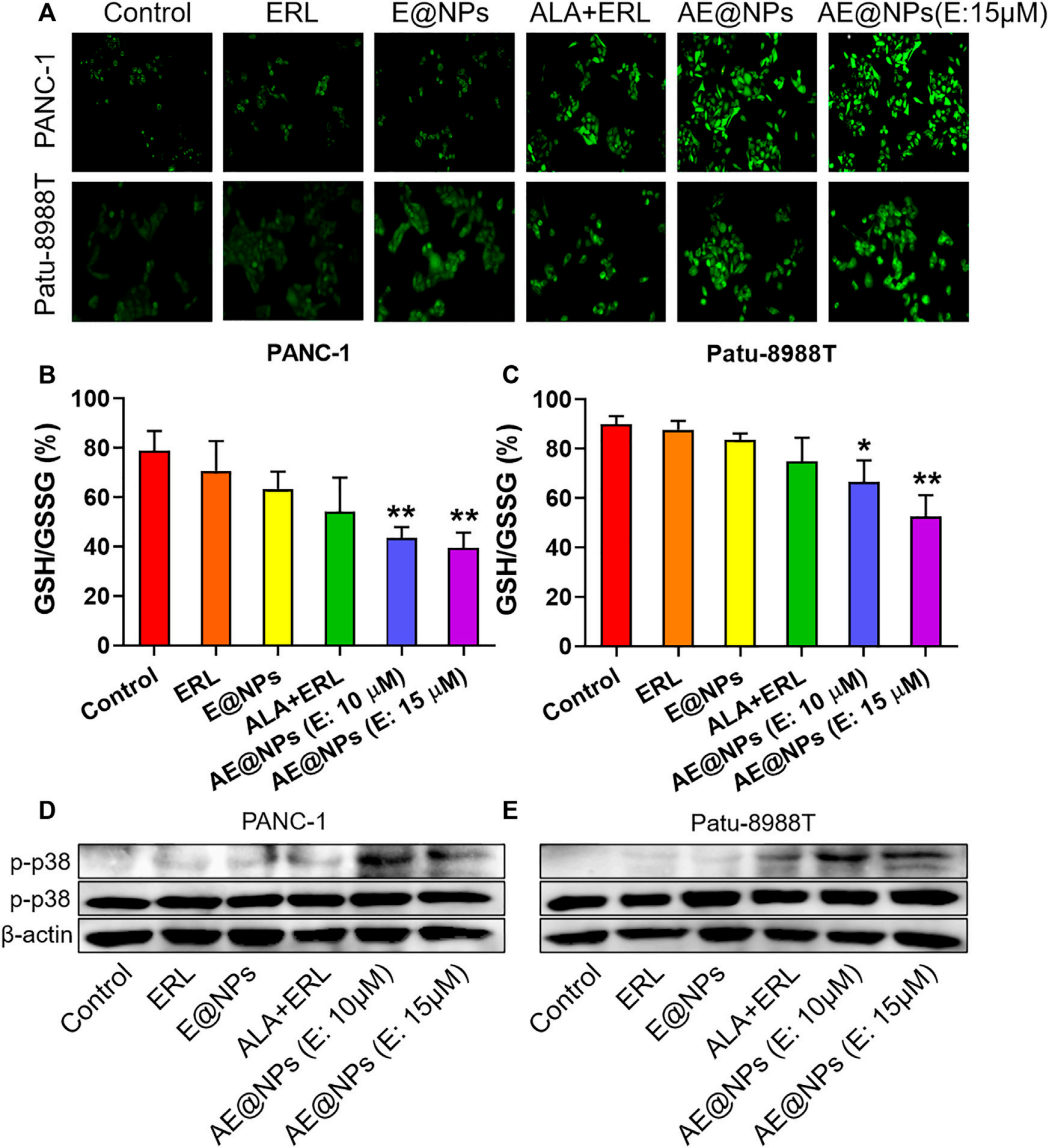
ROS-p38 axis was involved in the enhanced anticancer efficacy of AE@NPs **(A)** Representative images of cells stained with DCFH-DA to detect intracellular ROS generation (magnification: ×200). Quantification of GSH/GSSG ratio in **(B)** PANC-1 and **(C)** Patu-8988T cells after various treatments **(D)** PANC-1 and **(E)** Patu8988T were treated with various treatments for 18 h. The protein levels of p-p38, p38 were measured by western blot, with β-actin as an internal control. The data were shown as mean ± SD. *p < 0.05 **p < 0.01, compared to the control group.

ROS, including superoxide, hydroxyl radical, and hydrogen peroxide, is produced in all types of cells due to the incomplete electron transfer. Low levels of ROS could facilitate tumor initiation, development, and metastasis as signaling molecules, eliciting beneficial effects toward cancer cells. It is known that hydrogen peroxide could protect hypoxia inducible factor 1α (HIF-1α) from prolyl hydroxylase 2 (PHD2)-mediated degradation (Marinho et al., 2014); the stabilized HIF-1α level could promote angiogenesis, metabolic reprogramming, and metastasis of tumors. However, high levels of ROS could exert detrimental effects to tumor cells. As a matter of factor, chemotherapy and radiotherapy usually increase the ROS generation in tumor cells and make use of ROS-induced damage to kill the tumor cells (Kou et al., 2020b; Tang et al., 2020; Yang et al., 2020). It has been reported that ALA could induce apoptosis through upregulating ROS production along with GSH depletion in various cancers (Wang et al., 2018; Xu et al., 2019b; Yin et al., 2019). Therefore, we monitored the ROS level in PC cell after AE@NPs treatment using DCFH-DA. As shown in Figure 5A, the combination use of ERL and ALA significantly increased the ROS level as compared to ERL alone group, indicating ALA might contribute to the increased ROS production. The quantitative analysis of ROS generation was conducted by using flow cytometry (Supplementary Figure S3). The data were consistent with those shown in Figure 5A.

AE@NPs further improved the ROS production as compared to dual-drug solution group in both PANC-1 and Patu-8988T cells, which could be attributed to the increased uptake. The increased ROS level could exert enhanced anticancer treatment efficacy, which was consistent with the cell viability results (Figure 3). Glutathione is a key endogenous antioxidant and plays a critical role in the intracellular redox homeostasis of cancer cells. We further determined the ratio of GSH to oxidized GSH (GSSG) to evaluate the reductive capacity of PC cell after different drug treatments. As shown in Figures 5B,C, combined use of ALA and ERL slightly decreased the GSH/GSSG ratio as compared to ERL alone (*p* > 0.5). AE@NPs treatment showed a significantly decreased GSH/GSSG ratio, which might due to the increased uptake (Figure 2) and elevated ROS level (Figure 5A). Herein, we used drug combination of ERL and ALA codelivery by nanoparticles for enhanced anticancer therapy. Many studies showed that the high-level redox balance in cancer cells might contribute to the resistance of cancer cells to ERL therapy (Dong et al., 2018; Lei et al., 2019; Cheung et al., 2020). Besides inhibiting STAT3 activation, ALA might help elevate intracellular ROS level and scavenge reduced GSH, therefore further impairing the redox homeostasis and resulting in an enhanced anticancer efficacy. It has been confirmed that the activation of ROS-p38 axis could suppress the tumor growth, and even inhibit the proliferation of stem like cancer cells (Sato et al., 2014; Kou et al., 2019). Therefore, we studied the involvement of ROS responsive p38 MAPK signaling pathway to further understanding the molecular anticancer mechanisms of AE@NPs. As shown in Figures 5D,E, compared to other groups, the phosphorylation of p38-MAPK was increased after AE@NPs treatment, indicating the activation of ROS-p38 axis was also related with the enhanced anticancer therapy of AE@NPs. These findings suggest that the sensitivity of PC cells towards AE@NPs at least partially resulted from the activation of p38 MAPK pathway induced by GSH depletion and ROS production. It is well-acknowledged that *in vivo* studies are critical to evaluate the therapeutic efficacy and biosafety of the designed nanoparticles. In the following study, the *in vivo* behavior of AE@NPs would be assessed by pharmacokinetic study and biodistribution assay. PEGylated nanoparticles usually experienced prolonged circulation and tumor-specific accumulation by enhanced permeation and retention effect (Gabizon et al., 2020). In addition, the anticancer effect of AE@NPs would be systemically evaluated using tumor-bearing mice. Specifically, the biosafety of nanoparticles should be estimated by the changes of weight and serum biochemical indicators during drug treatment. ERL has been approved for clinic use in pancreatic cancer therapy for years; Inula helenium containing ALA has also long been used as a Chinese Herb Medicine for different therapeutic purpose and has recently been identified to possess anticancer activity. It could be expected that the combination use of ERL and ALA by nanoparticles could be applied to treat pancreatic cancer. Alone or together with surgery, radiotherapy, and immunotherapy, AE@NPs might achieve satisfactory therapeutic outcome, but require more systematic and detailed clinical studies.

## Conclusion

Acquired resistance occurrence negatively impact the clinical outcome of EGFR targeted therapies, and the activation of STAT3 pathway was believed to be responsible for that. To address this issue, we developed a nanoplatform to co-deliver ERL and ALA to inhibit both EGFR and STAT3 for enhanced PC treatment. The prepared ERL and ALA co-loaded nanoparticles (AE@NPs) possessed uniform size distribution and sustained drug release profile. AE@NPs showed increased cellular uptake in PC cells, resulting in enhanced cell-killing, increased anti-proliferation and anti-migration effects. Further mechanism study showed that AE@NPs markedly inhibited both EGFR and STAT3 signaling pathways and regulated Bax and Bcl-2 expression, therefore inducing distinct apoptosis. In addition, the activation of ROS-p38 axis also involved in the enhanced anticancer efficacy of AE@NPs. In conclusion, these results provided new insights into the anticancer activities and associated molecular mechanisms of AE@NPs, suggesting that such co-delivery treatment might potentially become a more effective combined drug therapy for PC.

## Data Availability

The original contributions presented in the study are included in the article/Supplementary Material, further inquiries can be directed to the corresponding authors.
