# Supplementary material for: Dual Targeting EGFR and STAT3 With Erlotinib and Alantolactone Co-Loaded PLGA Nanoparticles for Pancreatic Cancer Treatment
Source: Front Pharmacol. 2021 Mar 19;12:625084. doi: 10.3389/fphar.2021.625084 (PMC8017486; doi:10.3389/fphar.2021.625084)
Supplement: Supplementary file 1 [file datasheet1.docx]

Supplementary Material

**Table S1.** The pharmaceutical properties of NPs, E@NPs, and AE@NPs.

|  | Particle size (nm) | PDI | Zeta potential (mV) | EE of ERL (%) | DL of ERL (%) | EE of ALA (%) | DL of ALA (%) |
| --- | --- | --- | --- | --- | --- | --- | --- |
| NPs | 178.54 ± 4.56 | 0.109 ±0.029 | -5.69±1.13 |  |  |  |  |
| E@NPs | 182.69 ± 3.98 | 0.154 ± 0.036 | -4.88±0.87 | 90.25± 4.58 | 6.48±0.33 |  |  |
| AE@NPs | 185.97 ± 5.41 | 0.169 ± 0.044 | -5.23±0.54 | 88.69±6.61 | 6.38±0.47 | 91.24±3.27 | 3.27±0.12 |


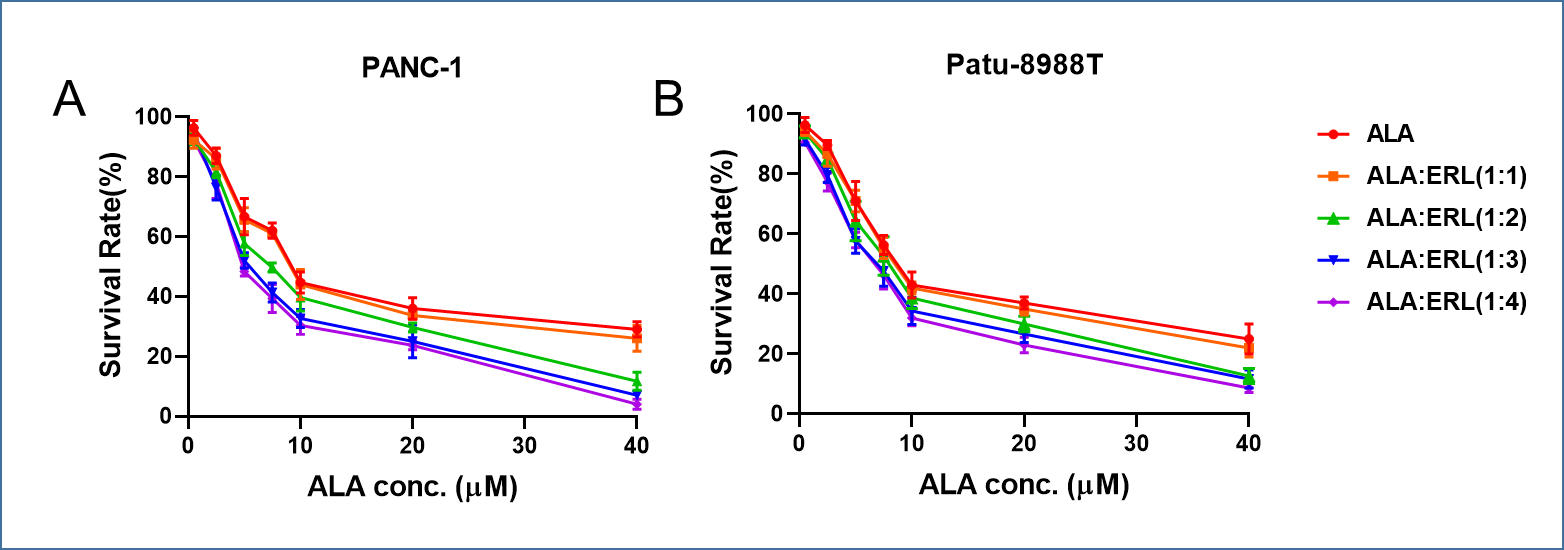
**Figure S1.** MTT assay was conducted to investigate the synergistic anticancer effect of ALA and ERL in (A) PANC-1 and (B) Patu-8988T cells. The results were shown as means ± SD (n=3).


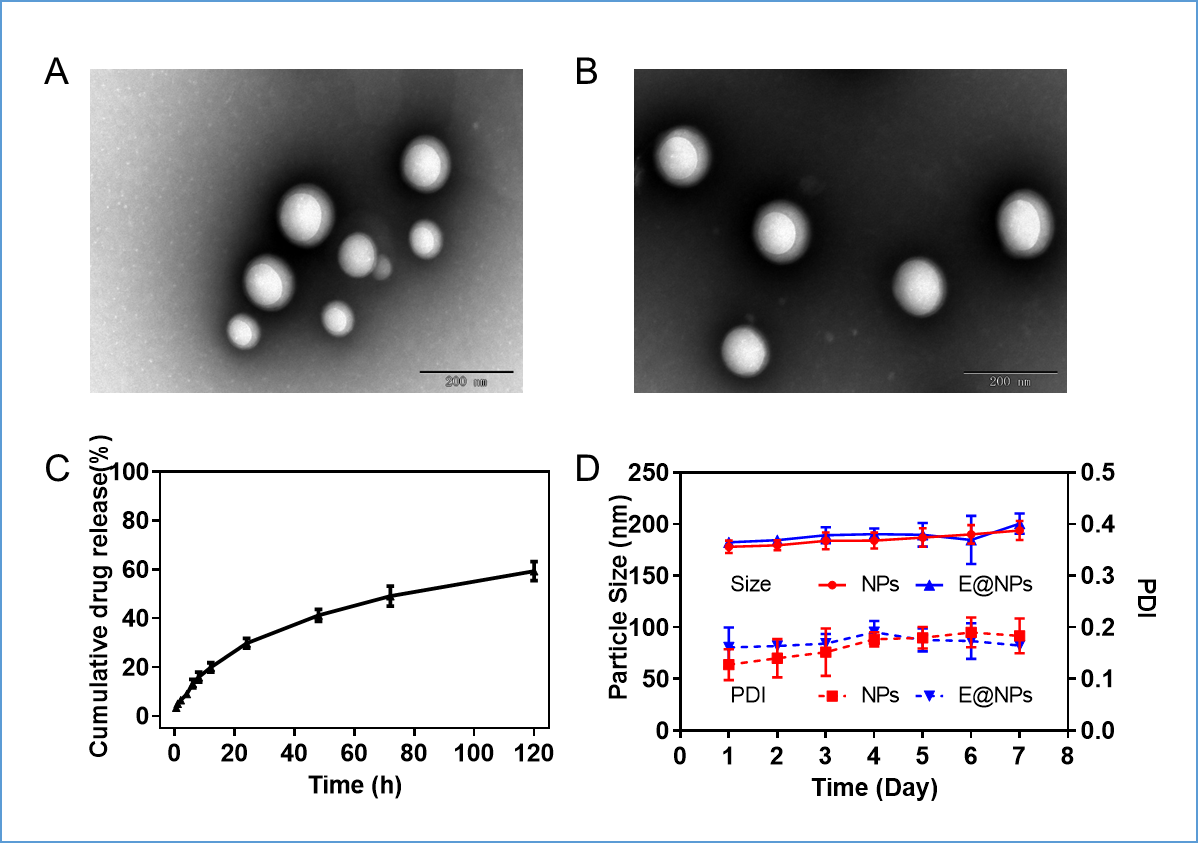


**Figure S2.** The physicochemical properties of NPs and E@NPs. The TEM images of (A) NPs and (B) E@NPs. (C) The cumulative ERL release from E@NPs. (D) The changes in particle size and PDI of NPs and E@NPs in pH 7.4 PBS at 4 °C in one week. The results were shown as means ± SD (n=3).


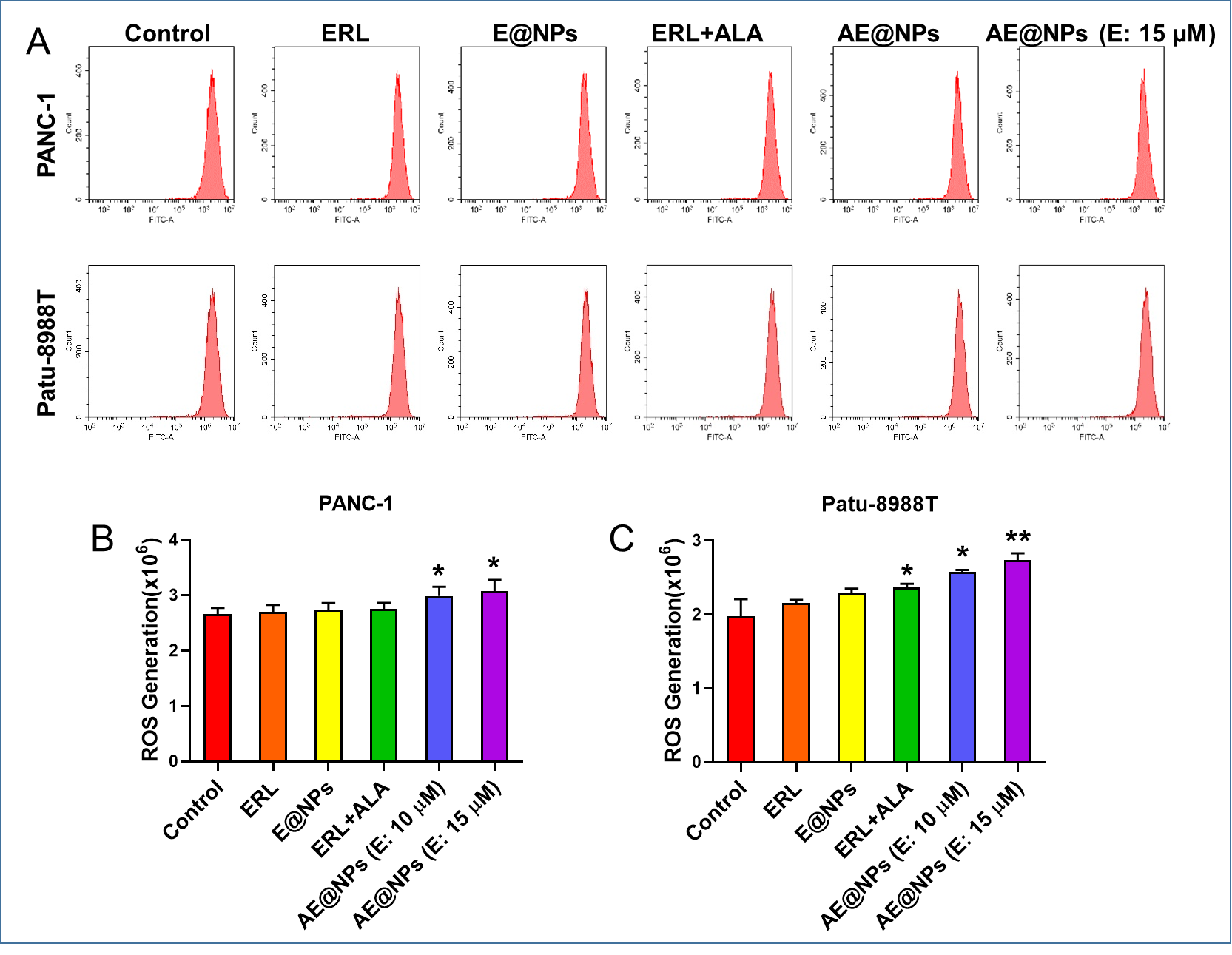


**Figure S3.** The ROS generation after different drug treatments in PC cells. (A) The ROS generation of PC cells after treatments was determined by flow cytometry. The values for ROS generation in (B) PANC-1 and (C) Patu-8988T cells after treatments. The data were shown as mean ± SD. Experiments were performed in triplicates. *P < 0.05 **P < 0.01, compared to the control group.
